# Supplementary material for: Spatial and Temporal Variations in PM10 Concentrations between 2010–2017 in South Africa
Source: Int J Environ Res Public Health. 2021 Dec 18;18(24):13348. doi: 10.3390/ijerph182413348 (PMC8706960; doi:10.3390/ijerph182413348)
Supplement: Supplementary file 1 [file ijerph-18-13348-s001.zip › ijerph-1482648-supplementary.pdf]

Table S1. The Pattern of PM_{10} data availability across the sites for year 2010–2017.

Province	Site	Site type	2010	2011	2012	2013	2014	2015	2016	2017
Gauteng	Bodibeng	Traffic			347(95%)	328(90%)				
	Booysen	Residential			326(89%)		291(80%)			
	Ekandustria	Industrial				261(72%)	309(85%)			
	Elandsfontein	Industrial							304(83%)	325(89%)
	Etwatwa	Residential			255(70%)					
	Germiston	Residential		293(80%)						
	Leandra	Industrial		293(80%)	275(75%)					
	Newtown	Residential			286(78%)					
	Olievenhoutbosch	Residential			334(91%)					
	Orange Farm	Residential	263(72%)							262(72%)
	Randwater	Industrial				305(84%)	359(98%)	300(82%)	336(92%)	278(76%)
	Rosslyn	Industrial			322(88%)	348(95%)	328(90%)			
	Tembisa	Residential		273(75%)						
	Thokoza	Residential		357(98%)						
	Watville	Residential			268(73%)					
Mpumalanga	Bosjesspruit	Industrial								332(91%)
	Camden	Industrial				349(96%)		298(81%)		302(83%)
	Chicken Farm	Industrial								349(95%)
	Club	Industrial			348(95%)	351(96%)	338(93%)		319(87%)	353(96%)
	Embalenhle	Industrial			296(81%)		311(85%)		333(91%)	328(90%)
	Ermelo	Industrial	334(92%)	347(94%)	321(88%)	301(82%)	309(85%)	279(76%)	260(71%)	
	Grootvlei	Industrial		359(98%)		340(93%)				
	Hendrina	Residential	286(78%)	284(78%)	292(80%)			303(83%)	340(93%)	
	Komati	Industrial		310(85%)	313(86%)		254(70%)	310(85%)	278(76%)	314(86%)
	Kriel village	Industrial								301(82%)
	Kwazamokuhle	Residential								290(79%)
	Middleburg	Industrial	299(82%)	270(74%)	346(95%)	334(92%)	306(84%)	343(94%)	299(82%)	
	Phola	Industrial				308(84%)	300(82%)		343(94%)	325(89%)
	Secunda	Industrial	317(87%)	293(80%)	264(72%)	338(93%)				

	Verykkop	Industrial				328(90%)			267(73%)	331(90%)
	Witbank	Residential	337(92%)			331(91%)	264(72%)	293(80%)	339(93%)	
Western Cape	Beliville	Industrial			301(82%)	348(95%)		338(92%)	305(83%)	396(81%)
	Foreshore	Traffic		284(78%)	325(89%)	344(94%)		284(76%)	354(97%)	329(90%)
	George	Traffic	358(98%)			259(71%)				
	Goodwood	Residential		346(95%)	339(93%)		318(87%)	286(78%)	349(93%)	
	Stellenbosch	Industrial			361(99%)	284(78%)				
	Tableview	Industrial		353(96%)	269(73%)	288(79%)				
	Wallacedene	Residential			337(92%)			298(81%)	334(91%)	354(97%)
KwaZulu-Natal	Brackenham	Residential		363(99%)	366(100%)			360(98%)	346(95%)	332(91%)
	CBD	Traffic		355(97%)	359(98%)	360(99%)		350(96%)	345(94%)	350(96%)
	City Hall	Traffic	342(94%)							
	Esikhaweni	Residential							358(98%)	326(89%)
	Ganges	Residential	277(76%)	344(94%)		307(84%)	292(80%)			
	Ferndale	Residential	330(90%)	305(83%)	256(70%)					
